# [Retracted] MicroRNA‑93 promotes cell proliferation by directly targeting P21 in osteosarcoma cells

**DOI:** 10.3892/etm.2024.12389

**Published:** 2024-01-15

**Authors:** Yu He, Bo Yu

Exp Ther Med 13:2003–2011, 2017; DOI: 10.3892/etm.2017.4204

Following the publication of the above paper, it was drawn to the Editors’ attention by a concerned reader that the GAPDH control western blotting data shown in Fig. 4C and D on p. 2008 were strikingly similar to data appearing in different form in other articles written by different authors at different research institutes that had either already been published elsewhere prior to the submission of this paper to *Experimental and Therapeutic Medicine*, or were under consideration for publication at around the same time. Owing to the fact that certain of these data had already apparently been published previously, the Editor of *Experimental and Therapeutic Medicine* has decided that this paper should be retracted from the Journal. The authors were asked for an explanation to account for these concerns, but the Editorial Office did not receive a reply. The Editor apologizes to the readership for any inconvenience caused.

